# Histone deacetylase 4 and 5 translocation elicited by microsecond pulsed electric field exposure is mediated by kinase activity

**DOI:** 10.3389/fbioe.2022.1047851

**Published:** 2022-11-17

**Authors:** Zahra Safaei, Gary L. Thompson

**Affiliations:** Department of Chemical Engineering, Rowan University, Glassboro, NJ, United States

**Keywords:** electroporation, breast cancer, calcium signaling, nucleocytoplasmic shuttling, HDAC

## Abstract

Electroporation-based technologies using microsecond pulsed electric field (µsPEF) exposures are established as laboratory and clinical tools that permeabilize cell membranes. We demonstrate a µsPEF bioeffect on nucleocytoplasmic import and export of enzymes that regulate genetic expression, histone deacetylases (HDAC) -4 and -5. Their μsPEF-induced nucleocytoplasmic transport depends on presence and absence of extracellular calcium ions (Ca^2+^) for both MCF7 and CHO-K1 cells. Exposure to 1, 10, 30 and 50 consecutive square wave pulses at 1 Hz and of 100 µs duration with 1.45 kV/cm magnitude leads to translocation of endogenous HDAC4 and HDAC5. We posit that by eliciting a rise in intracellular Ca^2+^ concentration, a signaling pathway involving kinases, such as Ca^2+^/CaM-dependent protein kinase II (CaMKII), is activated. This cascade causes nuclear export and import of HDAC4 and HDAC5. The potential of µsPEF exposures to control nucleocytoplasmic transport unlocks future opportunities in epigenetic modification.

## Introduction

Higher-order chromatin superstructure controls the basic cellular processes of transcription and replication. The fundamental chromatin building block in eukaryotic cells is comprised of multiple nucleosomal arrays of DNA packed around octamers of histone proteins. Nucleosome remodeling and post-translational modification of histones have evolved to control transcription. Histone modification *via* dynamic acetylation and deacetylation of the amino termini is regulated by histone acetyltransferase and deacetylase enzymes, respectively. Thus, these enzymes contribute to determining cell fate and behavior by modifying chromatin structure (Ayer, 1999).

Histone deacetylases (HDACs) are a group of enzymes that introduce epigenetic modifications. The HDACs’ major function is to take out an acetyl group from an ε-N-acetyl-lysine residue on a histone (Hsu et al., 2016). The HDAC enzyme group contains eighteen enzymes that are split into four classes- I, II, III, and IV (Halley et al., 2011). These classes are formed based on of expression patterns as well as structural and functional differences (Morris and Monteggia, 2013). Class IIa HDACs (including HDACs 4, 5, 7 and 9) are expressed in the cytoplasm and the nucleus and are able to shuttle between them (Jenke et al., 2021). This class of HDACs plays important roles in tissue growth and development, the regulation of gene transcription, and cell growth, survival, and proliferation. Their expression also is associated with cancer development (Backs et al., 2006; Li and Seto, 2016). Phosphorylation of these enzymes disrupts their interaction with transcription factors, promoting nuclear export and cytosolic accumulation and allowing activation of gene transcription to proceed. However, dephosphorylation of class IIa HDACs leads to nuclear import and easy access to specific transcription factors, enabling repression of genes (Paroni et al., 2008; Park and Kim, 2020).

In order to influence class IIa HDAC activities, a strategy to control their nuclear-to-cytoplasm shuttling is critical (Di Giorgio and Brancolini, 2016). HDAC levels vary based on the type of cell or tissue and the activity of upstream enzymes. Multiple enzymes within the kinase family such as CaMK enzymes, liver kinase B1 (LKB1)-dependent kinases of the 5′-adenosine monophosphate-activated protein kinase (AMPK) family, and protein kinase D (PKD) enzymes can phosphorylate class IIa HDACs (McKinsey et al., 2000b; Mihaylova et al., 2011; Mihaylova and Shaw, 2013). Phosphorylation prepares them for nuclear export *via* interaction with 14-3-3 adapter proteins (Grozinger and Schreiber, 2000; Wang et al., 2000; Kao et al., 2001). Conversely, dephosphorylation of these HDACs leads to their nuclear accumulation through breakage of their bond with 14-3-3 protein, enabling them to bind with HDAC3 located in the nucleus (Grozinger and Schreiber, 2000).

High voltage pulsed electric fields (PEF) with short durations have many applications in biological and medical science. The key feature of this technology ostensibly is creation of large pores within cellular membranes due to dielectric breakdown (Jordan et al., 1989). Electroporation technology is a well-established tool for gene electrotransfer including gene therapy by accurate plasmid delivery, drug delivery, and electrochemotherapy. As an FDA-approved clinical method, electroporation has delivered promising outcomes in cancer treatment, tumor ablation and DNA vaccination (Wong and Neumann, 1982; Chang, 1989; Mir et al., 1998; Neumann et al., 1999; Heller et al., 2017; Zhang et al., 2017; Aycock and Davalos, 2019). µsPEF exposure treats cancer by initiating cell death cascades, including apoptosis induction as result of electropermeabilization of biomembranes to small ions. A primary step in electropermeabilization cytoeffects is the rapid rise of intracellular calcium ion concentration, [Ca^2+^]_i_ (Cemazar et al., 1998; Mi et al., 2009; Chen et al., 2010; Ren and Beebe, 2011; Thompson et al., 2014).

Calcium ions play important roles within the cell, acting as messengers that control crucial cellular responses that affect apoptosis, muscle contraction, gene transcription, metabolism, etc. (Plaschke et al., 2019). Extracellular matrix (ECM), endoplasmic reticulum (ER), mitochondria and cytosol are the different sources of Ca^2+^ in cells. Typically, [Ca^2+^]_i_ is very small compared to that in the ECM. This small concentration is essential for communication and signaling processes to exist within the cell. The cellular response to exposure to µsPEF begins with electropermeabilization to small ions, especially Ca^2+^. Exposed cells theoretically develop ion-permeable nanopores in the plasma membrane (Pakhomov et al., 2007). These nanopores allow for an influx of Ca^2+^, which in turn alters mechanotransduction elements and sets off numerous signaling pathways linked to cell death and activation of different enzymes, as demonstrated in many cell lines (Estlack et al., 2014; Dai et al., 2017; Hanna et al., 2017). Furthermore, some studies report Ca^2+^-induced activation of protein phosphatase types 1, 2A and 2B (PP1, PP2A, PP2B) (Colbran, 2004), CaMKII (Prevarskaya et al., 2014) and cross-talk between Ca^2+^ and protein kinase A (PKA) (Howe, 2011; Melville et al., 2017).

The results of this study show that different repetitions of µsPEF exposure induce Ca^2+^ uptake and manipulate nucleocytoplasmic shuttling of class IIa HDACs, especially HDAC4 and HDAC5. These two HDACs have important roles in a variety of human cancers, such as breast, renal, bladder, colorectal and prostate cancer (Barneda-Zahonero and Parra, 2012; Sanaei and Kavoosi, 2019; Oltra et al., 2020; Jenke et al., 2021). We hypothesize that by eliciting an increase of [Ca^2+^]_i_ with µsPEF exposure, a signaling pathway involving CaMKII (Prevarskaya et al., 2014) and either PKA [by cross-talk with Ca^2+^ (Howe, 2011)] or AMPK (Salminen et al., 2016) is activated that leads to HDAC4 and HDAC5 nucleocytoplasmic shuttling. In unexposed MCF7 cells, treatment either with kinase inhibitor KN-93 or H-89 leads to phosphorylation of class IIa HDACs and subsequently their nuclear export. However, µsPEF exposure to 10 pulses induces nuclear accumulation of HDAC4 in which CaMKII affects nuclear accumulation while high [Ca^2+^]_i_ likely inhibits AMPK-based export of HDAC4 to the cytoplasm of the breast cancer cells. Interestingly, µsPEF exposure of CHO-K1 cells to 10 pulses displays opposite trends in which HDAC4 and HDAC5 do not undergo a significant amount of translocation unless CaMKII activity is inhibited *via* KN-93 treatment.

## Materials and methods

### Cells lines and reagents

Two common cell lines were used in this study. Chinese hamster ovary (CHO-K1; ATCC^®^ CCL-61™, Manassas, VA, United States) cells were grown in T-25 flasks which contained F12-K medium (Kaighn’s Modification of Ham’s F-12 Medium; ATCC^®^ 30-2004™, Manassas, VA, United States) supplemented with 10 vol% Fetal Bovine Serum (FBS, HyClone, SH30396.03, MA, United States) and 1 vol% Penicillin and Streptomycin (HyClone, SV30010, MA, United States). Cells were passaged with a ratio of 875 cells/µl into 6 ml complete media when cells reached about 70%–90% confluency. Cells were detached for splitting using 1 ml of 0.25 vol% Trypsin-EDTA solution (HyClone, MA, United States). The breast cancer MCF-7 (ATCC^®^ HTB-22™) cell line was grown in BD EMEM (Becton, Dickinson and Company, Sparks, MD, United States) (supplemented with 10 vol% FBS, 1 vol% Pen/Strep) with ratio of 378 cells/µl into 6 ml complete media. Insulin was not supplemented into the complete media. Cells were split as above, and cells were counted using an automated Revolutionary Science (Shafer, MN, United States) RevCount 150 cell counter. Cultures were incubated at 37°C, 5 vol% CO_2_, and 95% relative humidity. The range of passages used was between 6 and 12 times.

In order to track the influence of Ca^2+^, two kinds of custom buffer solutions were used throughout the experiments. Standard Outside Solution (SOS) consisted of 5 mM KCl, 2 mM CaCl_2_, 10 mM 4-(2-hydroxyethyl)-1-piperazineethanesulfonic acid (HEPES), 2 mM MgCl_2_, 10 mM Glucose, and 135 mM NaCl. Calcium-Free Standard Outside Solution (CAF) consisted of all the SOS components, except instead of CaCl_2_, it contained 2 mM potassium ethylene glycol tetra-acetic acid (K-EGTA). Solution pH was adjusted to 7.4 using NaOH.

### Cell staining

Both cell lines were trypsinized, pelleted, and resuspended in complete growth medium. 175 cells/μl CHO-K1 per sample or 37 cells/μl MCF7 per sample were plated on glass bottom petri dishes with 35 mm overall diameter and 10 mm glass diameter (Matsunami Glass, WA, United States). After 15 min, 3 ml media appropriate to each cell type was added to the petri dishes, which were then incubated overnight. Cells were counted by a Revolutionary Science (St. Paul, MN, United States) RevCount-150.

### Calcium Green assay

For the Calcium Green 1-AM assay (CaGr, Cayman Chemical Company # 20400, Ann Arbor, MI, United States), 2 mM of CaGr was prepared by dilution in dimethyl sulfoxide (DMSO). On days of experiments, 1 µM working solution was prepared in phosphate buffered saline (PBS) using the lowest probe concentration to preclude overloading toxicity. The concentration of Ca^2+^ was determined empirically. To prepare the samples of CHO-K1 and MCF-7 cells, 50 µl of the working solution were added to each sample and incubated for 15 min at room temperature in the dark. The samples were then washed with 3 ml PBS to remove excess probe that either was not loaded or not associated with the membrane before imaging. To track the movement of Ca^2+^, confocal fluorescence microscopy images were acquired at 1 or 2 frame-per-second (fps) over 200 frames, using the microscope system described below.

### Enzyme inhibition

50 μM water-soluble KN-93 (BioVision # 1909, Milpitas, CA, United States) and 2 μM H-89 dihydrochloride (AdipoGen, San Diego, CA, United States) were used to inhibit CaMKII and other basophilic kinases (AMPK, PKA, etc.), respectively. To inhibit these enzymes in cells, cells were incubated in 2 ml of inhibitor diluted in full serum media for 1 h before μsPEF exposure. Cells were then incubated in 1 ml of inhibitor diluted in CAF or SOS during and 2 h after pulse exposure.

### Immunofluorescence assay for HDAC4 and HDAC5

To track endogenous HDAC4 and HDAC5 localization, each sample was fixed with 200 μl of 4% paraformaldehyde for 20 min at room temperature. Samples were then washed three times with 2 ml PBS–one time immediately and two times with a 15 min incubation. To permeabilize the membranes, 1 ml of 1 vol% Triton X-100 in PBS (PBSTx) was then added to the cells with an incubation lasting 15 min. Next, 200 μl of blocking solution, consisting of 5 w/v% of bovine serum albumin diluted in PBSTx solution, was applied to the samples for 60 min. Afterward, 50 μl of 5 μg/ml HDAC4 polyclonal antibody (BioVision # 3604A-100, Milpitas, CA, United States) or 20 μg/ml HDAC5 antibody (BioVision # 3605-100, Milpitas, CA, United States) were introduced to the fixed cell samples and incubated at 4°C overnight. Notably, the HDAC4 antibody we used for IFA recognizes human HDAC4 at amino acid 10, which lies within the nuclear localization sequence of HDAC4. These samples were then washed three times with 2 ml of 0.1 vol% Tween-20 in PBS (PBT) for 30 min. The secondary antibody and fluorescence marker was goat anti-rabbit IgG (H&L) (DyLight™ 488, NC, United States); 50 μl of which was diluted in blocking solution (1:500), added onto each sample, and incubated at 4°C overnight in the dark. Samples were then washed two times with 2 ml PBT with an incubation of 30 min.

### Propidium iodide nucleic acid stain

For detecting nuclei, samples were washed two times with 2 ml of 2XSSC solution consisting of 0.3 M NaCl and 0.03 M sodium citrate with pH 7.0. For the purpose of removing all released RNA, 1 ml of 100 μg/ml of Ribonuclease A (VWR #E866, Solon, OH, United States) in 2XSSC was added to each sample and incubated at 37°C for 20 min. Samples were then washed with 2 ml 2XSSC–three times fast and once with a 4 min incubation. Finally, 300 μl PI (1:500 dilution in 2XSSC) were loaded for 30 min in each sample, followed by three rinses with 2XSSC before imaging.

### Pulse treatment

Before pulse treatment, each sample petri dish was filled with 1 ml of SOS or CAF. Square wave pulses were generated by a BTX Gemini X2 electroporation system (Holliston, MA, United States) and delivered *via* a BTX Petri Dish Pulser with thirteen gold-plated electrodes arrayed so that each pair was 2 mm apart. For Ca^2+^ uptake, cells were pulsed with either 1 or 10 consecutive pulses with a duration of 100 µs each at a repetition rate of 1 Hz. The 100 µs duration of each pulse was chosen based on prior publications that investigated Ca^2+^ influx into CHO-K1 cells (Thompson et al., 2014) and breast cancer cell lines (Hanna et al., 2017) following PEF exposure. Furthermore, an FDA-approved clinical technique based on irreversible electroporation, Angiodynamics NanoKnife, uses 100 µs duration pulses. The 1 Hz pulse repetition rate for multiple pulse exposures was chosen for stability among successive pulses (limiting droop) and to allow sufficient time between pulses for dissipation of any heat generated by Joule heating.

The electrical resistance of the sample (including electrode array, bathing solution, and cells) was measured at imaging frame 20 using a relatively small (15 V) pre-pulse, and the μsPEF exposure was delivered at frame 40. The applied voltages tested were 100, 200 and 300 V, with a “sham” control of 0 V. For determining translocation of HDACs, cells were pulsed with 300 V with 1, 10, 30 and 50 consecutive pulses with a duration of 100 µs each and a repetition rate of 1 Hz. The total specific energy input, *W*_*S*_, provided to a sample during μsPEF exposure depends on numerous factors as represented by Eq. 1:

$$W_{S}=\frac{V^{2}*t_{p}*n}{R*m}$$ (1)

where *V* is the applied voltage, *t*_*p*_ is the pulse duration, *n* is the number of pulses, *R* is the measured electrical resistance, and *m* is the mass of the sample. In order to provide enough time for HDAC localization, samples were fixed 2 h after exposure.

### Microscopy imaging

Imaging was performed using a Thorlabs Confocal Microscopy Upgrade (Newton, NJ, United States) attached to an Olympus IX-73 microscope (Tokyo, Japan). For HDAC translocation and viability experiments, the Olympus UPlanFLN 40x/N.A. 1.30 oil objective was used. Fluorophore excitation was induced using 488 and 642 nm solid-state lasers. Emission was detected using 525 nm (±25 nm) and 670 nm (±20 nm) filter sets. Images were acquired at different positions of each sample, including the center and at least four opposing corners of the glass bottom with 1.0 fps and a 200 µm pinhole size. The size of images was 2048 × 2048 pixels.

Typically, a cross-pattern of five pairs of images (each pair consists of one channel of nuclear stain and one channel of HDAC immunofluorescence) was acquired from each sample petri dish. (Some petri dishes exhibited uneven distribution of cells, and images with little to no cells were omitted from analyses.) The five pairs of images were captured in a specific pattern: middle, upper right, lower right, lower left, and then upper left (Supplementary Table S1). A range of 4–9 images from one petri dish was captured per experimental condition (Supplementary Table S2). A total of 182 images representing 32 conditions for CHO-K1 cells were analyzed, and a total of 247 images representing 36 conditions for MCF7 cells were analyzed. This corresponded to a median and standard deviation of 6 ± 0.808 images per condition for CHO-K1 cells and 6 ± 0.996 images per condition for MCF7 cells. The images had a mean area covered by 44.36 ± 5.302% of CHO-K1 cells or 42.93 ± 9.163% of MCF7 cells.

Image analysis was performed on the entirety of masked regions within a given image instead of single cells because of the different morphological characteristics of MCF7 and CHO-K1 cells. MCF7 cells grew into clusters, whereas CHO-K1 cells tended to retain some separation until confluent. It was difficult to precisely distinguish individual MCF7 cell boundaries within clusters using the IFA channel. Therefore, trends in N/C ratios calculated from raw integrated densities normalized to percent area covered by cells are compared. CHO-K1 cell densities that gave similar percent area covered by cells per image as for MCF7 samples were used (Supplementary Table S2). The images produced were processed using the Fiji distribution of ImageJ2 (Schindelin et al., 2012) (Supplementary Table S1).

### Statistical analyses

Statistical analyses were performed with GraphPad Software (San Diego, CA, United States) Prism 9 using either the one-way ANOVA with the Dunnet posttest, two-way ANOVA with Tukey test, where appropriate. Results are shown in plots with significance determined as (ns) *p* < 0.1234, *p < 0.0332, **p < 0.0021, ***p < 0.0002 and ****p < 0.0001. Error bars in the presented graphs represent one standard deviation.

### COMSOL multiphysics simulation

**FIGURE 1:**
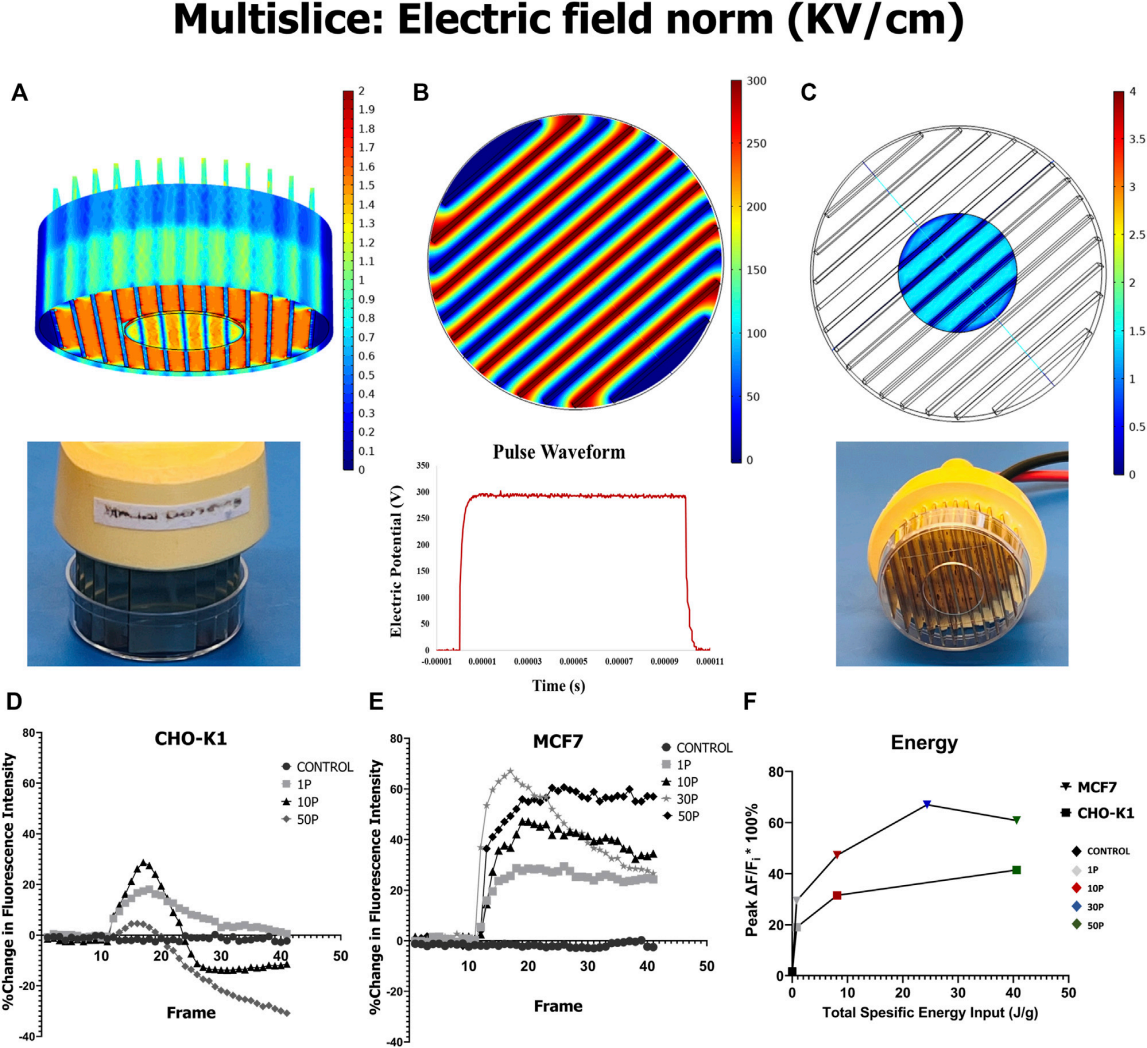
COMSOL model of µsPEF exposure, and Ca^2+^ uptake and release within cells elicited by µsPEF exposure. **(A)** A contour view of a 3D model of the electrode array illustrates the distribution of the electric field within the exposure media in the petri dish and at the glass bottom surface. **(B)** A multislice view of the same electrode array shows electric potential between each pairing of electrodes. **(C)** A corresponding multislice view emphasizes the electric field distribution in the media at the glass bottom to which the cells are attached. **(D)** Mean fluorescence intensity of CaGr per time from CHO-K1 cells and from **(E)** MCF7 breast cancer cells is plotted as a moving average of every five consecutive data points. A µsPEF exposure of 10 pulses each with 100 µs duration has been delivered at a repetition rate of 1 Hz. **(F)** For a given total specific energy input by µsPEF exposure, MCF7 cells experience a greater absolute change in peak fluorescence intensity than CHO-K1 cells.

The COMSOL (Burlington, MA, United States) Multiphysics^®^ (ver. 5.4) AC/DC module was used in a 3D model. The model contained two major domains. One of them included electrode subdomains which cover the thirteen gold-plated electrodes, and the other domain associated the glass-bottom subdomain (Figure 1). Cells were assumed to be attached to and thus received the same level of exposure as the glass subdomain. The color scale represented an electric field strength in kV/cm, with lower magnitudes indicated by darker blue and higher values shown as warmer colors at the circular glass slide.

## Results

### COMSOL model

This COMSOL model determines the electric field distribution within the 1 ml of bath solution (SOS or CAF) covering the cells adhered onto the glass-bottom slide (Figure 1). Cells in regions between pairs of electrodes are exposed to an electric field strength of approximately 1.45 kV/cm when a pulse of 300 V is delivered (Figure 1C). This electric field strength applied during a single 100 µs duration square wave pulse (Figure 1B, bottom) is on the same order of magnitude as reported to induce an increase in [Ca^2+^]_i_ in the entire population of Chinese hamster lung cells (CD-3F) and human adipose mesenchymal stem cells (haMSC) (Hanna et al., 2017). Therefore, such an exposure is expected to have similar effects on CHO-K1 and MCF7 cells.

### Cytosolic Ca^2+^ concentration following microsecond pulsed electric field exposure

To confirm the rise of [Ca^2+^]_i_ in response to µsPEF exposure, cells are loaded with CaGr, and its fluorescence intensity is monitored continuously before and after pulse delivery. By first finding the threshold voltage and number of pulses to significantly increase [Ca^2+^]_i_, it is predicted that these same conditions can be used to initiate downstream signaling cascades. Applied voltages <300 V do not result in significant rises in [Ca^2+^]_i_ in either cell type given a single pulse (data not shown); so, the numbers of pulses is varied at 300 V (Figures 1D–F). Mean fluorescence intensity of CaGr indicates relative [Ca^2+^]_i_ following PEF exposure of 100 µs duration and different numbers of pulses delivered at 1 Hz pulse repetition rate. For both MCF7 and CHO-K1 cells, a greater number of pulses leads to higher absolute peak change in [Ca^2+^]_i_ (Figures 1D,E). For a given total specific energy input, MCF7 cells experience a greater change in [Ca^2+^]_i_ than CHO-K1 cells (Figure 1F).

### Histone deacetylases localization following microsecond pulsed electric field exposure

**FIGURE 2:**
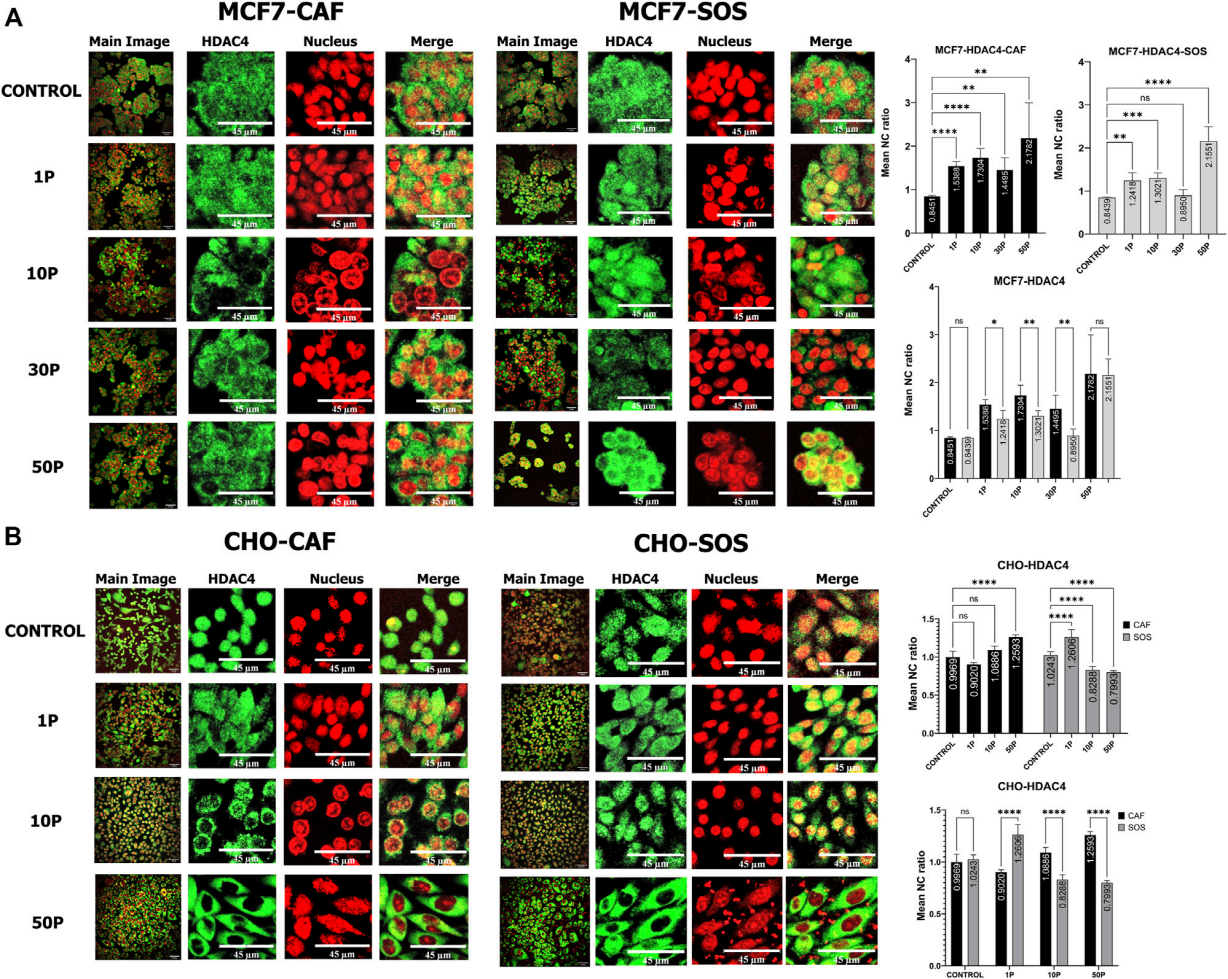
HDAC4 translocation within MCF7 and CHO-K1 cells elicited by µsPEF exposure. Immunostaining of HDAC4 (green) in **(A)** MCF7 cells or **(B)** CHO-K1 cells in either CAF or SOS solution. Each sample was exposed to 0 (control), 1, 10, 30 or 50 consecutive pulses, P, of 100 µs duration, 1.45 kV/cm and a repetition rate of 1 Hz. Nuclei are stained with PI (red). Image contrast has been enhanced for complete visualization of the boundaries of cells and nuclei. The representative Main Image shows the full-view from which a zoomed-in area is selected for the two HDAC and Nucleus images to the right of the Main Image. Graphs show comparisons of mean nuclear to cytoplasmic ratio (NC ratio) of HDAC4 in unexposed controls versus treatments with different pulse numbers in CAF and SOS. Each exposure in CAF and SOS is compared. Data represent 5–9 images from one dish per condition (Supplementary Table S2). Statistical significance tested by ANOVA is indicated as (ns) *p* < 0.1234, *p < 0.0332, **p < 0.0021, ***p < 0.0002 and ****p < 0.0001.

**FIGURE 3:**
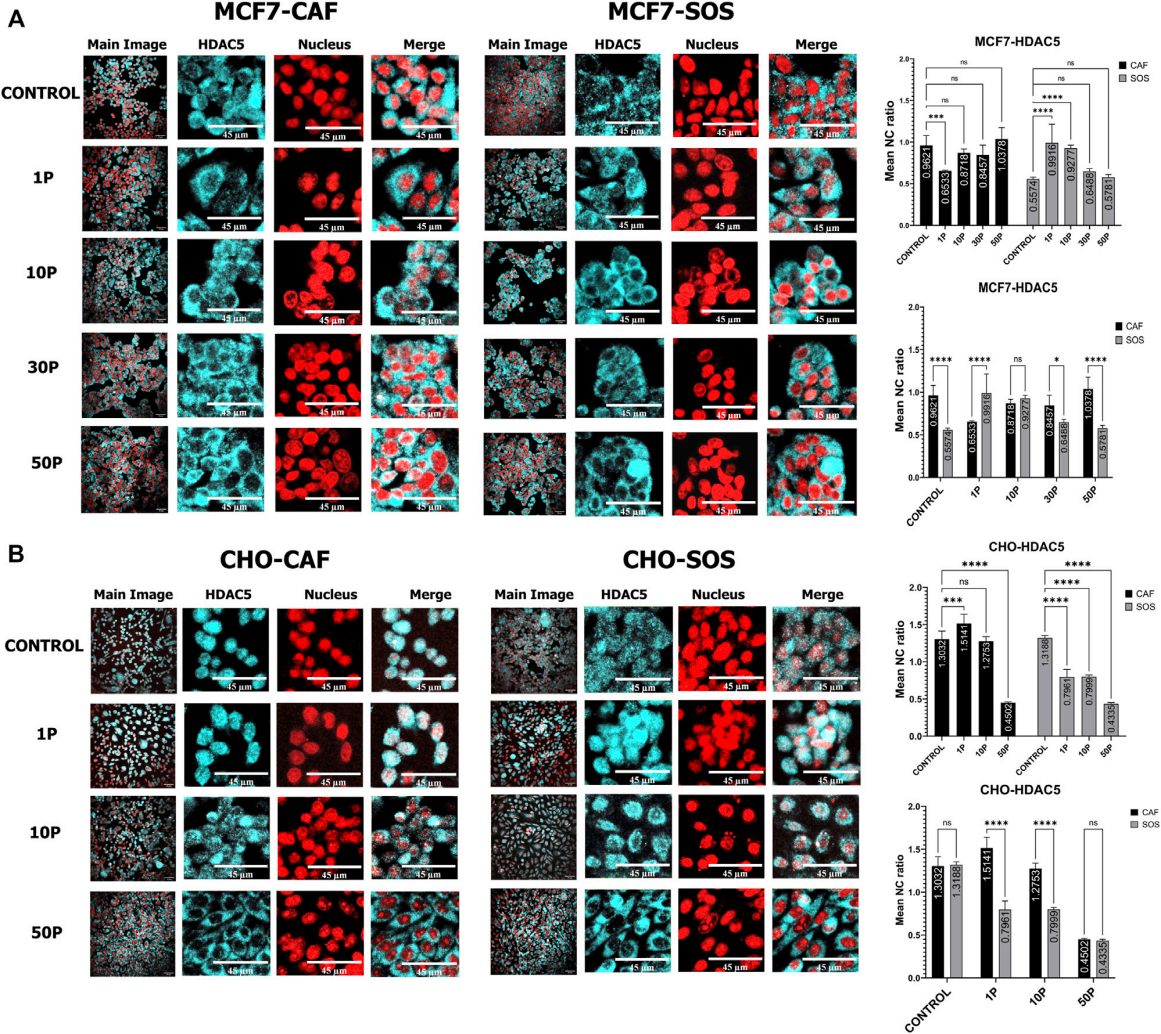
HDAC5 translocation within MCF7 and CHO-K1 cells elicited by µsPEF exposure. Immunostaining of HDAC5 (cyan) in **(A)** MCF7 cells or **(B)** CHO-K1 cells in either CAF or SOS solution. Each sample was exposed to 0 (control), 1, 10, 30 or 50 consecutive pulses, P, of 100 μs duration, 1.45 kV/cm and a repetition rate of 1 Hz. Nuclei are stained with PI (red). Image contrast has been enhanced for complete visualization of the boundaries of cells and nuclei. The representative Main Image shows the full-view from which a zoomed-in area is selected for the two HDAC and Nucleus images to the right of the Main Image. Graphs show comparisons of mean nuclear to cytoplasmic ratio (NC ratio) of HDAC5 in unexposed controls versus treatments with different pulse numbers in CAF and SOS. Each exposure in CAF and SOS is compared. Data represent 4–8 images from one dish per condition (Supplementary Table S2). Statistical significance tested by ANOVA is indicated as (ns) *p* < 0.1234, *p < 0.0332, **p < 0.0021, ***p < 0.0002 and ****p < 0.0001.

To observe the effect of µsPEF exposure on nucleocytoplasmic shuttling of endogenous HDAC4 and HDAC5 in MCF7 and CHO-K1 cells, immunofluorescence images of cells with and without exposures are compared (Figures 2, 3). For MCF7 cells, µsPEF exposure leads to nuclear accumulation of HDAC4 within 2 h of exposure, both in the presence (in SOS) and absence (in CAF) of extracellular Ca^2+^ (Figure 2A). In either solution, the mean nuclear-to-cytoplasmic ratio (N/C ratio) of HDAC4 exhibits increasing nuclear accumulation when exposed to 1, 10, and 50 pulses. The trend in nuclear accumulation in MCF7 cells is lowest in CAF and statistically insignificant in SOS given 30 pulses. The N/C ratios are significantly greater in CAF than SOS for 1, 10, and 30 pulse exposures of MCF7 cells.

For HDAC5 in MCF7 cells (Figure 3A), higher µsPEF exposure does not lead to significant translocation within 2 h of exposure, both in SOS (for 10, 30, and 50 pulses) and CAF (30 and 50 pulses). Yet, 1 pulse in CAF leads to HDAC5 export from the nucleus, whereas 1 and 10 pulses in SOS lead to nuclear import. The mean N/C ratios of HDAC5 in CAF versus SOS are significantly different for 1 pulse but are not different given 10 pulses for MCF7 cells.

In CHO-K1 cells, the presence of extracellular Ca^2+^ plays a significant role in HDAC4 translocation (Figure 2B). No significant translocation occurs in CAF after exposure to 1 and 10 pulses. In contrast, 1 pulse in SOS leads to nuclear accumulation, and 10 pulses in SOS lead to cytoplasmic accumulation. Exposure to 50 pulses in SOS also results in cytoplasmic accumulation, whereas 50 pulses in CAF causes nuclear accumulation.

The relative trends in changes of N/C ratios of HDAC4 in response to µsPEF exposure of MCF7 and CHO-K1 cells is consistent with preliminary results (Supplementary Figure S1), despite lower concentration of BSA in the blocking solution in these preliminary samples.

In CHO-K1 cells, the presence of extracellular Ca^2+^ also plays a significant role in HDAC5 translocation in response to µsPEF exposure with 1 and 10 pulses (Figure 3B). In CAF, no significant translocation occurs after exposure to 10 pulses, but 1 pulse leads to nuclear accumulation whereas 50 pulses cause significant cytoplasmic accumulation. By contrast in SOS, all levels of µsPEF exposures induce cytoplasmic accumulation of HDAC5. The mean N/C ratios in CAF versus SOS are similar for sham and 50 pulse exposures. These data indicate that in the presence of extracellular Ca^2+^, µsPEF exposures up to a threshold number of pulses (at most 50 pulses) elicit cytoplasmic accumulation of HDAC5 in CHO-K1 cells.

The presence of Ca^2+^ in the bathing solution increases the probability of activating Ca^2+^-dependent enzymes that are key modifiers of nucleocytoplasmic shuttling of HDAC4. Our data indicate significant mitigation of nuclear accumulation in MCF7 cells exposed to 1, 10, and 30 pulses when bathed in SOS. This mitigation effect does not appear in MCF7 cells exposed to 50 pulses–significant nuclear accumulation is similar in both solutions given the highest total specific energy input used (Figure 2A). Nuclear accumulation also is mitigated in CHO-K1 cells exposed to 10 and 50 pulses when bathed in SOS (Figure 2B). However, exposure of CHO-K1 cells to a single pulse in SOS leads to increased nuclear accumulation of HDAC4.

### Connection of kinase activities to histone deacetylases localization

**FIGURE 4:**
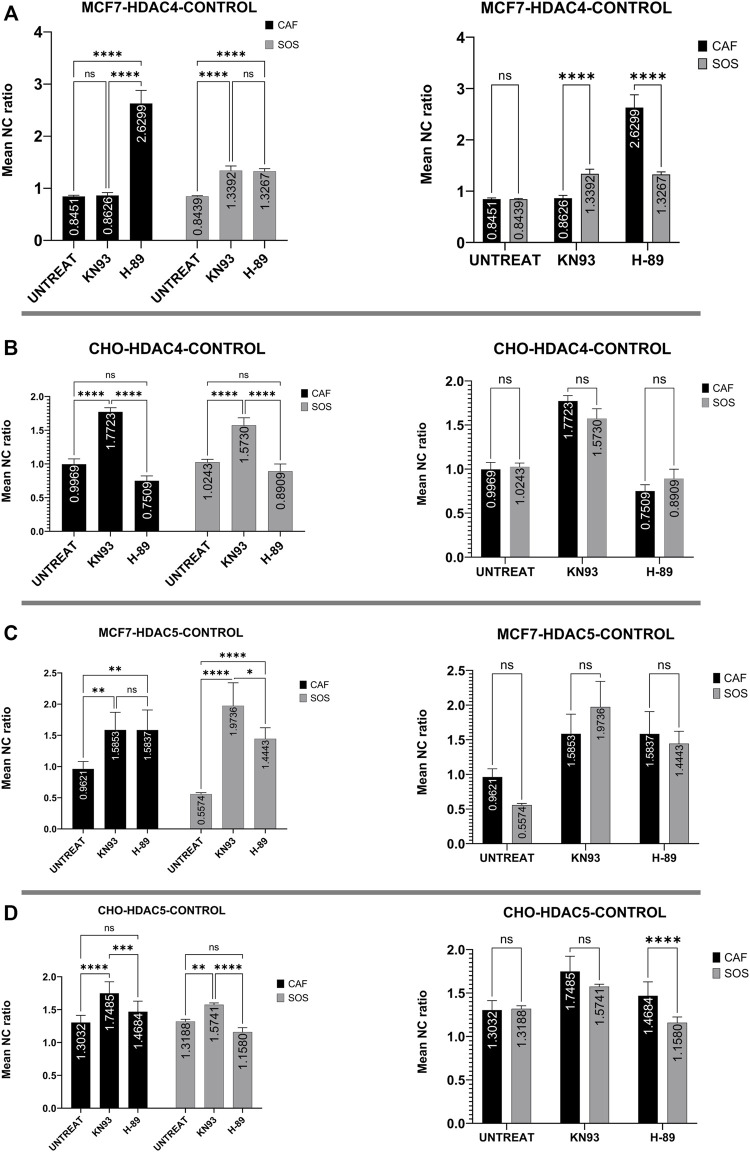
Kinase inhibitor effects on HDAC4 and HDAC5 localization in unexposed (i.e., no µsPEF exposure) MCF7 and CHO-K1 cells. Unexposed, untreated (UNTREAT) cells are compared with KN-93 or H-89 treated cells in either CAF or SOS (left). The significance of extracellular Ca^2+^ is tested for each condition (right). Data represent 5–6 images from one dish per condition (Supplementary Table S2). Statistical significance tested by ANOVA is indicated as (ns) *p* < 0.1234, *p < 0.0332, **p < 0.0021, ***p < 0.0002 and ****p < 0.0001. **(A)** HDAC4 in MCF7, **(B)** HDAC4 in CHO-K1, **(C)** HDAC5 in MCF7, **(D)** HDAC5 in CHO-K1.

To identify the relative contribution of different kinases to endogenous HDAC4 and HDAC5 translocation, the mean N/C ratios of HDAC4 or HDAC5 with respect to the presence of the selected kinase inhibitors are compared (Figure 4). These comparisons do not involve any µsPEF exposures. We first want to know the specific effects of these kinases on HDAC4 and HDAC5 localization within cells after 2 h of pharmacological treatment.

MCF7 cells treated with a common basophilic kinase inhibitor, H-89, showed significantly higher nuclear accumulation of HDAC4 in both SOS and CAF. H-89 significantly inhibits PKA, AMPK and some other basophilic kinases (Davies et al., 2000; Limbutara et al., 2019). In CAF, CaMKII inhibition by KN-93 treatment of MCF7 cells failed to alter the mean N/C ratio as compared to control samples with no pharmacological inhibitor. In SOS, by contrast, KN-93 inhibition caused significant nuclear accumulation compared to controls. Activation of CaMKII by Ca^2+^ modifies AMPK-based nuclear export of HDAC4 (Park et al., 2011), equalizing the export activity of these kinases. Therefore, our results suggest CaMKII and AMPK are at least partially responsible for exporting HDAC4 from nucleus to cytoplasm in MCF7 cells in a Ca^2+^-dependent manner, and AMPK has a dominant role in this phenomenon (Figure 4A).

However, in CHO-K1 cells, only CaMKII enzymes appear significantly responsible for nuclear export of HDAC4, whereas H-89 fails to alter nucleocytoplasmic transport of HDAC4. In CHO-K1 cells, KN-93 and H-89 application do not reveal any significant Ca^2+^-dependence of these kinases’ influence on HDAC4 localization (Figure 4B).

In MCF7 cells, KN-93 and H-89 treatment affect HDAC5 localization similarly (Figure 4C). Separate treatment with these inhibitors shows that both sets of kinases contribute to shuttling HDAC5 from nucleus to cytoplasm. In CAF, inhibition by these drugs leads to similar mean N/C ratios of HDAC5. Within SOS, CaMKII inhibition by KN-93 results in a significantly higher mean N/C ratio than for kinase inhibition using H-89. However, the effects of each inhibitor on HDAC5 localization are independent of extracellular Ca^2+^ in MCF7 cells.

In CHO-K1 cells, only KN-93 treatment significantly affects HDAC5 localization, leading to nuclear accumulation independently from extracellular Ca^2+^ (Figure 4D). Inhibition by H-89 fails to alter the mean N/C ratio from that of uninhibited control CHO-K1 cells.

### Alteration of microsecond pulsed electric field exposure-induced histone deacetylases translocation by inhibition of kinases

**FIGURE 5:**
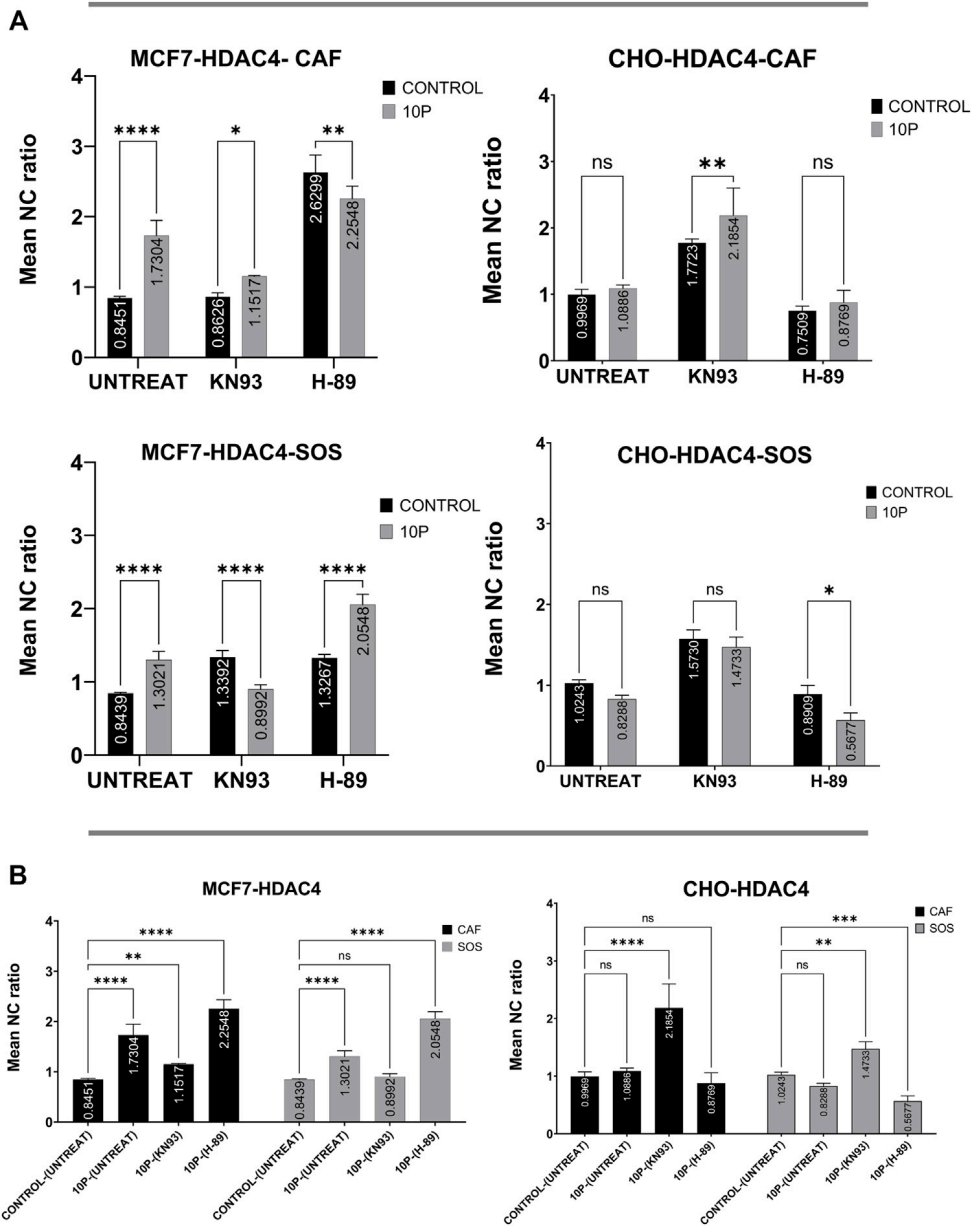
Kinase inhibitor effects on HDAC4 translocation with and without µsPEF exposure of cells. **(A)** Comparisons of N/C ratios between unexposed control samples and samples exposed to 10 pulses of µsPEF for MCF7 (left) and CHO-K1 (right) cells in CAF (top) and SOS (middle) show that MCF7 cells have different responses in all conditions, whereas the responses of CHO-K1 are the same in all but two conditions. **(B)** Comparisons between untreated, unexposed samples and exposed or exposed and treated conditions in CAF and SOS. Data represent 5 – 6 images from one dish per condition (Supplementary Table S2). Statistical significance tested by ANOVA is indicated as (ns) *p* < 0.1234, *p < 0.0332, **p < 0.0021, ***p < 0.0002 and ****p < 0.0001.

**FIGURE 6:**
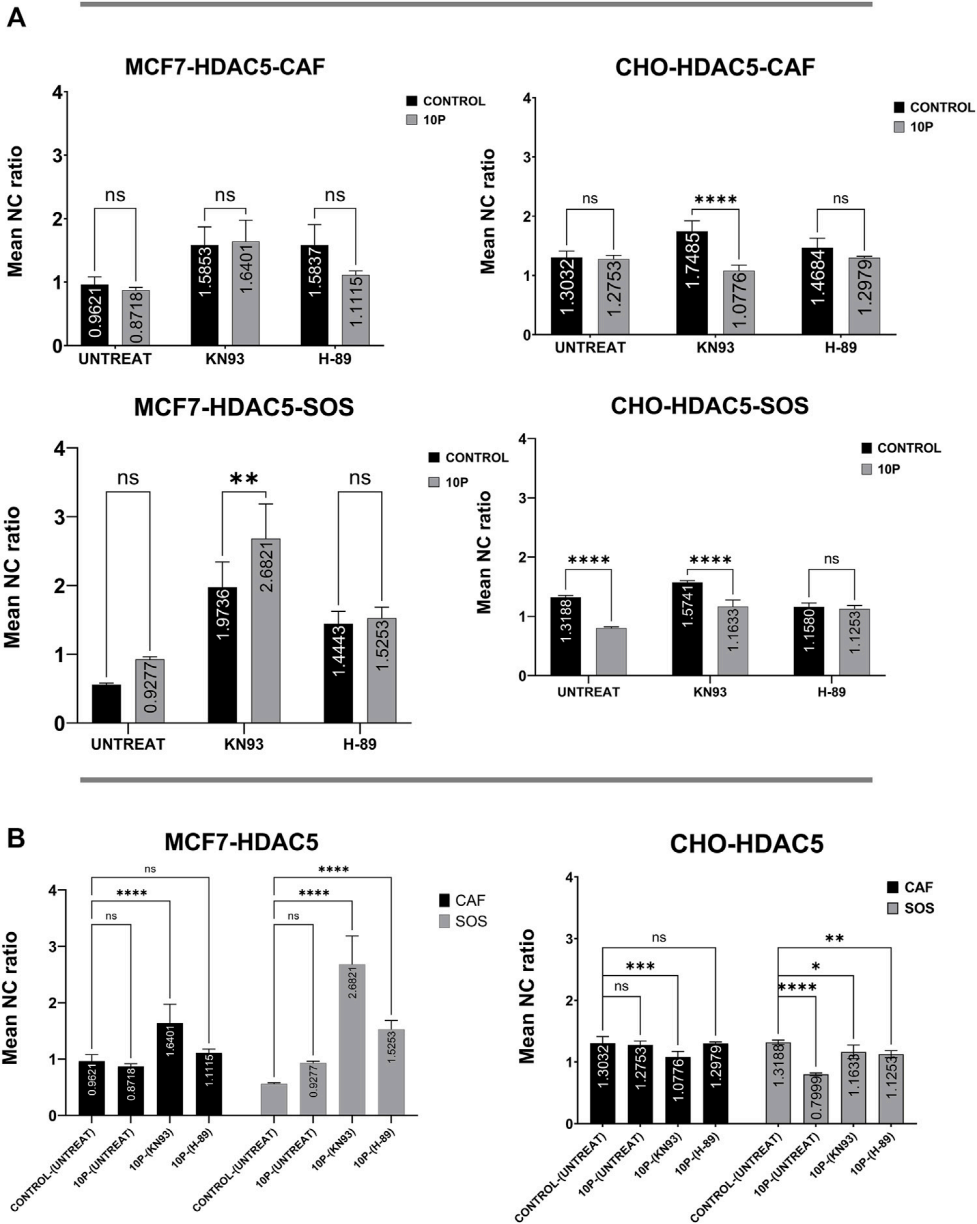
Comparison of kinase inhibitor effects on HDAC5 translocation with and without µsPEF exposure of cells. **(A)** Comparisons of N/C ratios between unexposed control samples and samples exposed to 10 pulses of µsPEF for MCF7 (left) and CHO-K1 (right) cells in CAF (top) and SOS (middle) show that MCF7 cells only has a significant rise in nuclear accumulation of HDAC5 following µsPEF exposure in SOS with KN-93. Conversely, CHO-K1 experiences less nuclear localization of HDAC5 in both SOS and CAF with KN-93. **(B)** Comparisons between untreated, unexposed samples and exposed or exposed and treated conditions in CAF and SOS. Data represent 5–6 images from one dish per condition (Table S2). Statistical significance tested by ANOVA is indicated as (ns) *p* < 0.1234, *p < 0.0332, **p < 0.0021, ***p < 0.0002 and ****p < 0.0001.

To determine whether inhibition of these kinases impact µsPEF exposure-induced translocation of endogenous HDAC4 and HDAC5, we compare sham exposure samples to samples exposed to 10 pulses of µsPEF (Figures 5, 6). Each pair of samples is bathed in SOS or CAF and contains: no inhibitor, H-89, or KN-93.

In MCF7 cells, the main effect of µsPEF exposure is nuclear accumulation of HDAC4, except when KN-93 is applied in SOS and when H-89 is used in CAF (Figure 5 left). Relative to the control sham samples without inhibitors, µsPEF exposure overall results in significant nuclear accumulation of HDAC4 (N/C ratio >1.0), except when KN-93 is in SOS. In both SOS and CAF, µsPEF exposure-induced nuclear accumulation of HDAC4 is enhanced by H-89 treatment in the breast cancer cell line.

For HDAC5 in MCF7 cells, the combination of KN-93 and µsPEF exposure in SOS accrues more HDAC5 in the nucleus than with KN-93 treatment alone (Figure 6 left). There is no significant change in HDAC5 localization from µsPEF exposure in the presence of H-89. Relative to sham exposure samples without inhibitors, µsPEF exposure does not significantly change HDAC5 localization unless combined with CaMKII inhibition (in both CAF and SAS) or H-89 treatment (in SOS), which leads to nuclear accumulation.

For HDAC4 in CHO-K1 cells, µsPEF exposure only leads to nuclear accumulation of HDAC4 when KN-93 is used (Figure 5 right). In SOS, more cytoplasmic accumulation occurs in response to µsPEF exposure when H-89 is used.

In CHO-K1 cells, µsPEF exposure in SOS leads to cytoplasmic accumulation of HDAC5, as does µsPEF exposure combined with CaMKII inhibition in CAF and SOS (Figure 6 right). As for MCF7 cells, there is no significant change in HDAC5 localization from µsPEF exposure when H-89 is used. Relative to sham exposure samples without inhibitors, µsPEF exposure in SOS causes significant cytoplasmic accumulation.

### Roles of kinases in microsecond pulsed electric field exposure-induced histone deacetylases translocation

**FIGURE 7:**
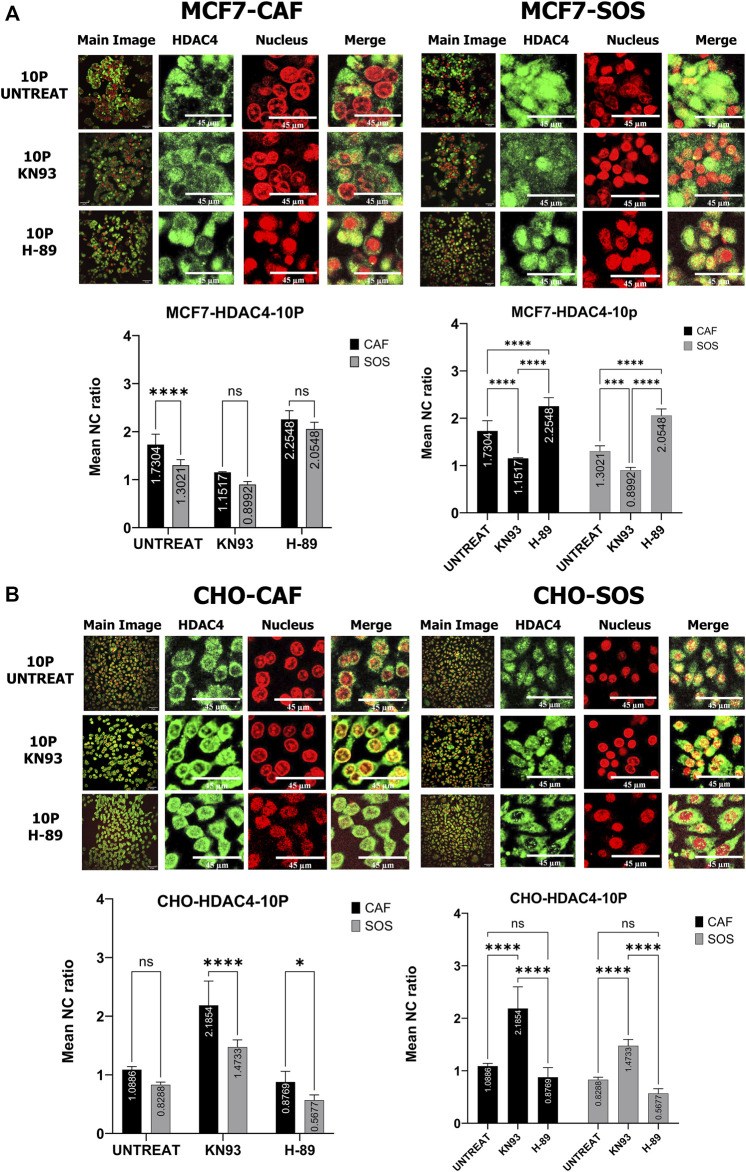
Kinase inhibitor effects on HDAC4 translocation within MCF7 and CHO-K1 cells elicited by µsPEF exposure. Representative confocal fluorescence images show HDAC4 (green) localization relative to nuclei (red) within **(A)** MCF7 or **(B)** CHO-K1 cells exposed to 10 pulses of µsPEF without or in the presence of KN-93 or H-89 in CAF or SOS. Image contrast has been enhanced for complete visualization of the boundaries of cells and nuclei. The representative Main Image shows the full-view from which a zoomed-in area is selected for the two HDAC and Nucleus images to the right of the Main Image. Mean NC ratios of HDAC4 in exposed cells without or in the presence of KN-93 or H-89 are compared between CAF and SOS bathing solutions to determine the effect of extracellular Ca^2+^ on the N/C ratios. The mean N/C ratios of HDAC4 in exposed, untreated cells in CAF or SOS are compared between those in the presence of KN-93 or H-89 to determine the effect of inhibitors on the response of cells to µsPEF exposure. Data represent 5–6 images from one dish per condition (Table S2). Statistical significance tested by ANOVA is indicated as (ns) *p* < 0.1234, *p < 0.0332, **p < 0.0021, ***p < 0.0002 and ****p < 0.0001.

To better understand the Ca^2+^-dependent association of these kinases with HDAC4 during µsPEF exposure-induced translocation, statistical comparisons among µsPEF exposure (10 pulses) samples in SOS and CAF are made (Figure 7). In MCF7 cells responding to µsPEF exposure, the results suggest that CaMKII is important for nuclear accumulation of HDAC4, since the mean N/C ratios in the presence of KN-93 are significantly lower than for uninhibited, exposed samples (Figure 7A). H-89 treatment indicates PKA or AMPK participates conversely in export of HDAC4 from the nucleus. Presence of extracellular Ca^2+^ in SOS decreases the mean N/C ratios of all exposed samples, indicating a mitigation of nuclear accumulation (Figure 7A right). However, this decrease is only significant in uninhibited samples, and the contributions of CaMKII, PKA and AMPK to HDAC4 translocation are independent of extracellular Ca^2+^ (Figure 7A left). These trends are consistent with AMPK-mediated export of HDAC4 from the nucleus and CaMKII-associated nuclear accumulation of HDAC4 in response to this level of µsPEF exposure of MCF7 cells.

The contributions of these kinases to the translocation of HDAC4 within CHO-K1 cells exposed to µsPEF are dissimilar to those of MCF7 cells’ responses (Figure 7B). In CHO-K1 cells responding to µsPEF exposure, inhibition of CaMKII activity leads to enhanced nuclear import of HDAC4 in both SOS and CAF, while H-89 treatment does not play a significant role. The presence of Ca^2+^ impacts the effectiveness of CaMKII inhibition, resulting in a reduced N/C ratio in response to µsPEF exposure.

**FIGURE 8:**
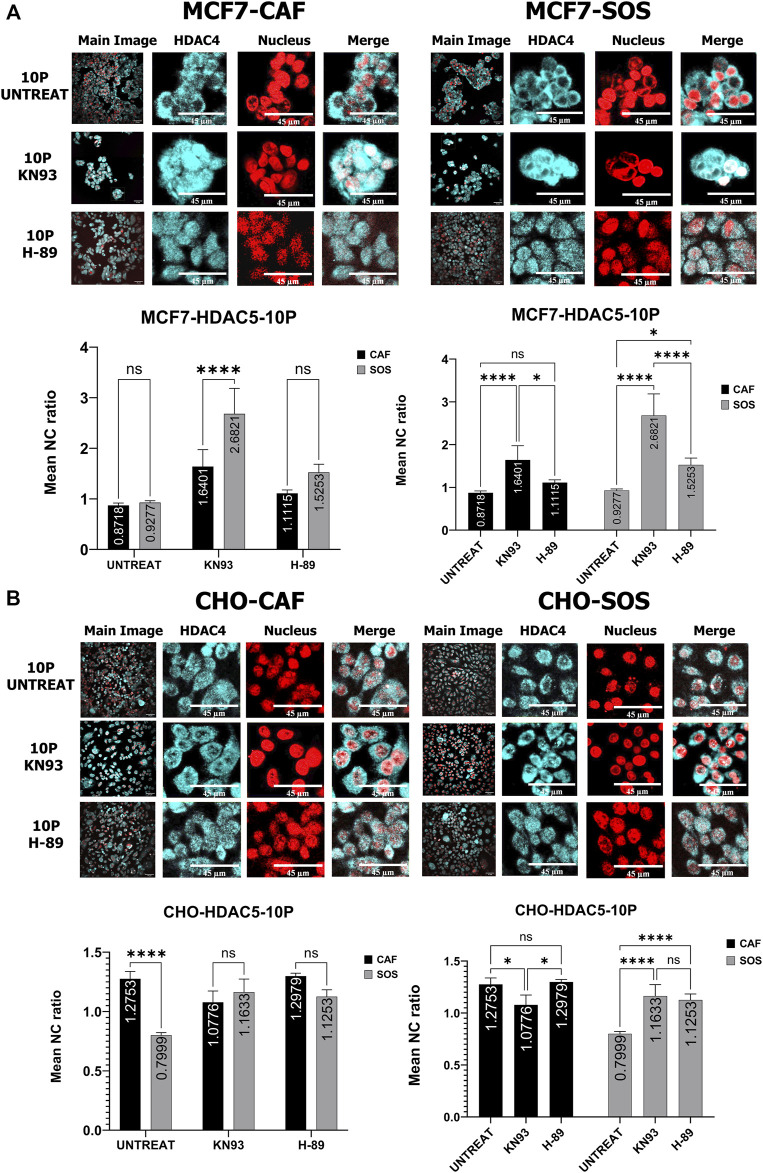
Kinase inhibitor effects on HDAC5 translocation within MCF7 and CHO-K1 cells elicited by µsPEF exposure. Representative confocal fluorescence images show HDAC5 (cyan) localization relative to nuclei (red) within **(A)** MCF7 or **(B)** CHO-K1 cells exposed to 10 pulses of µsPEF without or in the presence of KN-93 or H-89 in CAF or SOS. Image contrast has been enhanced for complete visualization of the boundaries of cells and nuclei. The representative Main Image shows the full-view from which a zoomed-in area is selected for the two HDAC and Nucleus images to the right of the Main Image. Mean N/C ratios of HDAC5 in exposed cells without or in the presence of KN-93 or H-89 are compared between CAF and SOS bathing solutions to determine the effect of extracellular Ca^2+^ on the N/C ratios. The mean N/C ratios of HDAC5 in exposed, untreated cells in CAF or SOS are compared between those in the presence of KN-93 or H-89 to determine the effect of inhibitors on the response of cells to µsPEF exposure. Data represent 5 – 6 images from one dish per condition (Supplementary Table S2). Statistical significance tested by ANOVA is indicated as (ns) *p* < 0.1234, *p < 0.0332, **p < 0.0021, ***p < 0.0002 and ****p < 0.0001.

Considering HDAC5 translocation in MCF7 cells responding to µsPEF exposure, the results show that inhibition of CaMKII in either solution and H-89 in SOS lead to greater N/C ratios (Figure 8A). Therefore, both CaMKII and AMPK control µsPEF exposure-induced export of HDAC5 from the nucleus in a Ca^2+^-dependent manner, with CaMKII dominating this role.

For HDAC5 in CHO-K1 cells responding to µsPEF exposure, CaMKII and AMPK impact for HDAC5 nuclear export in SOS, whereas in CAF, CAMKII inhibition conversely leads to a lesser N/C ratio of HDAC5 (Figure 8B). Interestingly, µsPEF exposure in SOS without inhibitors decreases the N/C ratio and causes cytoplasmic accumulation of HDAC5. Inhibition using KN-93 or H-89 prevents cytoplasmic accumulation of HDAC5 in CHO-K1 cells responding to µsPEF exposure in SOS.

## Discussion

**FIGURE 9:**
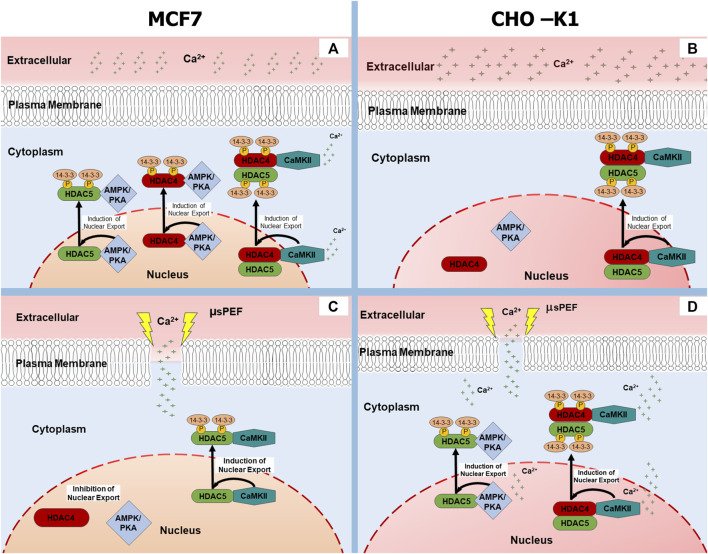
Kinase-based regulation of HDAC4 and HDAC5 nucleocytoplasmic shuttling in MCF7 (left) and CHO-K1 (right) cells. When Ca^2+^ is shown adjacent to a molecule, its action is observed only in SOS. Otherwise, the actions depicted occur in both SOS and CAF. **(A)** Unexposed MCF7 cell, **(B)** Unexposed CHO-K1 cell, **(C)** MCF7 cell exposed to µsPEF, **(D)** CHO-K1 cell exposed to µsPEF.

Clarifying the mechanisms that enact HDAC4 and HDAC5 localization induced by µsPEF exposure of mammalian cells has significant translational implications due to the pervasive roles of class IIa HDAC’s in cancer (Clocchiatti et al., 2011; Miller et al., 2011). We demonstrate that µsPEF exposure-induced translocation of HDAC4 and HDAC5 depends upon kinase activity, and that this translocation is cell type-dependent. Figure 9 shows our proposed models for µsPEF exposure-induced HDAC4 and HDAC5 translocation in a breast cancer cell line, MCF7, as compared to a cell line, CHO-K1, commonly used in electrophysiology (Gamper et al., 2005). As supported by data in Figure 5, µsPEF exposure leads to cytoplasmic accumulation of HDAC5 within CHO-K1 cells in SOS, whereas in MCF-7 cells, µsPEF exposure elicits significant levels of nuclear accumulation of HDAC4. We observe these changes in HDAC localization within 2 h of µsPEF exposure, which is too short a time window for upregulated expression. This combined with the impact of kinases on HDAC4 and HDAC5 localization following µsPEF exposure suggests nucleocytoplasmic shuttling occurs.

Nucleocytoplasmic shuttling of endogenous class IIa HDAC’s has been indicated in a variety of mammalian cells, including: cardiomyocytes (Liu et al., 2020; Helmstadter et al., 2021), hepatocytes (Mihaylova et al., 2011), macrophages (Wang et al., 2022), endothelial cells (Liu et al., 2017), and neurons (Kassis et al., 2015). Depending on cell and tissue type, intracellular localization of HDAC4 and HDAC5 plays a role in the physiology of: long-term memory formation (Wang et al., 2011; Schlumm et al., 2013; Main et al., 2021), angiogenesis (Liu et al., 2017), glycogen storage (Mihaylova et al., 2011), and cell proliferation (Guan et al., 2012). Here we observe localization of endogenous HDAC’s in adherent human and hamster cell lines. The differential responses of HDAC localization in these cell types to stimuli from pharmacological and µsPEF exposures is not unexpected, given cellular subtype specific expression patterns of HDAC’s and their distinct and dynamic roles in processes such as differentiation and cancer development (Mathias et al., 2015; Yang et al., 2021).

Electrical and pharmacological stimuli are known to induce shuttling of HDAC4 and HDAC5. Electrical pacing of skeletal muscle fibers (Liu et al., 2005; Liu and Schneider, 2013) and of cardiomyocytes (Ljubojevic et al., 2014; Karppinen et al., 2018; Helmstadter et al., 2021) regulates HDAC4 and HDAC5 localization *via* [Ca^2+^]_i_-dependent activation of CaMKII and PKA. In these studies, electrical stimuli are only characterized as pulses of 1 ms duration delivered in trains with repetitions between 0.2 and 10 Hz. There is no indication of electric field strength, pulse waveform, or whether pulses are monophasic or biphasic. But it may be presumed that these myocyte stimulation experiments do not encroach upon electroporation field strength thresholds, and rises in [Ca^2+^]_i_ are attributable to voltage-gated ion channels opening, activation of receptors, and release of internal stores (Karppinen et al., 2018). Increasing the frequency in this range of myocyte pacing leads to higher [Ca^2+^]_i_, greater amounts of active phosphorylated CaMKII, and thus more HDAC4 efflux from the nucleus (Liu et al., 2005; Ljubojevic et al., 2014). Inhibition of CaMKII activation using the pharmacological antagonist KN-93 effectively blocks electrical pacing-induced nuclear efflux of HDAC4 (Liu and Schneider, 2013; Helmstadter et al., 2021). However, in resting skeletal muscle fibers, CaMKII inhibition by KN-62 did not significantly alter HDAC4 localization, whereas application of the broader-spectrum kinase antagonist, staurosporine, blocked HDAC4 translocation (Liu et al., 2005). Therefore, a variety of kinases affect localization dynamics of HDAC4 in resting and stimulated myocytes.

PKA has been identified as the main antithesis to CaMKII in directing HDAC4 translocation in myocytes. Agonists that phosphorylate and activate PKA direct nuclear influx of HDAC4 in skeletal muscle fibers and cardiomyocytes. Pretreatment with inhibitors of PKA mitigated induction of HDAC4 nuclear influx (Liu and Schneider, 2013). During electrical pacing of healthy cardiomyocytes, PKA-dependent nuclear accumulation of HDAC4 predominate during the initial 10 min of response, while CaMKII-driven nuclear efflux becomes dominant beyond 10 min as active CaMKII accrues. In pre-hypertrophic failing cardiomyocytes, the kinetics of this co-regulation shift so that CaMKII-dependent nuclear efflux dominates even at earlier times (Helmstadter et al., 2021).

Our results suggest such a co-regulation between CaMKII and another kinase inhibited by H-89 exists in a [Ca^2+^]_i_-dependent manner in unexposed MCF7 cells but not CHO-K1 cells. The N/C ratio of HDAC4 in MCF7 breast cancer cells without exposure to µsPEF (Figure 4A) partially reflect the mechanisms of HDAC4 translocation described above for myocytes. As depicted in Figures 9A,B, both CaMKII and kinases inhibited by H-89 are responsible for cytoplasmic accumulation of HDAC4 in the presence of extracellular Ca^2+^, whereas CaMKII does not exhibit a significant role in HDAC4 translocation in the absence of Ca^2+^. H-89-inhibited kinases appear to dominate HDAC4 translocation within MCF7 cells, especially in CAF. However, in CHO-K1 cells, only inhibition of CaMKII alters the N/C ratio of HDAC4 regardless of extracellular [Ca^2+^], and H-89-inhibited kinases appear insignificant for its translocation. Under normal culture conditions, MCF7 and CHO-K1 regulate HDAC4 localization *via* opposing mechanisms, and they maintain differential responses to µsPEF exposure.

MCF7 cells respond to electrical stimulus in the form of µsPEF exposure with HDAC4 translocation more readily than CHO-K1 cells (Figures 5, 9). The general mammalian cell response to exposure begins with electropermeabilization to small ions, especially Ca^2+^. Exposed cells theoretically develop ion-permeable nanopores in the plasma membrane (Pakhomov et al., 2007). According to our data in Figure 1, exposure to 10 square-wave pulses of 100 µs duration at 1.45 kV/cm increases [Ca^2+^]_i_. Furthermore, it has been reported that shorter nsPEF exposures release intracellular Ca^2+^ stores due to nanoporation of subcellular membranes. Although we did not control for intracellular or intranuclear release of Ca^2+^, the CAF bath conditions would reduce [Ca^2+^]_i_ rises while µsPEF exposure causes release of intracellular Ca^2+^ stores (Buescher and Schoenbach, 2003; White et al., 2004; Schoenbach et al., 2017). Thus, influx of extracellular Ca^2+^ plus intracellular Ca^2+^ release produce our observed [Ca^2+^]_i_. Notably, CHO-K1 cells express a dearth of voltage-gated ion channels (Gamper et al., 2005), whereas like myocytes, MCF7 cells express a range of voltage-gated ion channels (Berzingi et al., 2016). For a given total specific energy input from µsPEF exposure (Figure 1F), CHO-K1 experience less of a change in [Ca^2+^]_i_ than MCF7 cells.

Effectively, µsPEF exposure acts as a signal that initiates Ca^2+^-dependent signalling cascades within mammalian cells. Immediately downstream of the increase in [Ca^2+^]_i_ is calmodulin (CaM), a Ca^2+^-dependent protein found in the cytoplasm. When each binding site or lobe of CaM is saturated with Ca^2+^, it undergoes a conformational change that permits CaM to interact with and activate a diverse set of enzymes, most importantly CaMKII (Prevarskaya et al., 2014). CaMKII phosphorylates specific domains of HDAC4, creating binding sites for 14-3-3 chaperone protein, which excludes nuclear import of HDAC4 (Figure 9). HDAC4 can subsequently enter the nucleus after dephosphorylation and dissociation from 14-3-3. And it should be noted that KN-93 not only inhibits CaMKII but also can block voltage-gated K^+^ channels and L-type Ca^2+^ channels (Gao et al., 2006; Pellicena and Schulman, 2014; Hegyi et al., 2015; Johnson et al., 2019), potentially lowering [Ca^2+^]_i_ changes induced by µsPEF exposure.

**FIGURE 10:**
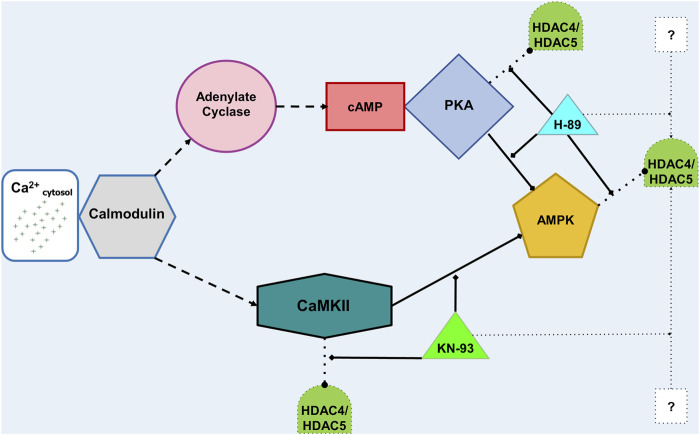
Diagram linking cytosolic Ca^2+^ concentration to HDAC4 and HDAC5 translocation. Cytosolic Ca^2+^ activates CaM, which goes on to activate (dashed lines) adenylate cyclase (AC, top) or CaMKII (bottom). On the top path, stimulated AC produces cAMP, which activates PKA. PKA can both act on (dotted lines) HDAC’s *via* phosphorylation and dephosphorylation. Activated PKA also phosphorylates and thus inhibits (solid lines) AMPK. If AMPK is active in the nucleus, it can phosphorylate HDAC’s. H-89 can inhibit PKA and AMPK. On the bottom path, activated CaMKII can phosphorylate HDAC’s and inhibit AMPK. KN-93 can inhibit CaMKII. Finally, both H-89 and KN-93 can affect other proteins, denoted by the question marks (?), which may impact intracellular HDAC localization.

Active CaM also stimulates adenylyl cyclase (AC) to increase production of cyclic adenosine monophosphate (cAMP), which activates PKA (Figure 10). Active PKA in the nucleus can phosphorylate HDAC4 at Ser-740, leading to nuclear export (Backs et al., 2008; Shimizu et al., 2014). There also are multiple amino acid sites along HDAC4 that PKA dephosphorylates. Although athermal nsPEF exposure can inactivate the catalytic activity of the PKA-C subunit in solution (Beebe, 2015), more often PKA is reported as promoting nuclear accumulation of HDAC4, as described above for skeletal muscle myocytes and cardiomyocytes (Backs et al., 2006; Liu and Schneider, 2013).

Among the other basophilic kinases that H-89 inhibits (Davies et al., 2000; Limbutara et al., 2019), nuclear AMPK can phosphorylate HDAC4 and HDAC5, leading to their binding to 14-3-3 protein and export from the nucleus (Salminen et al., 2016; Niu et al., 2017) (Figure 9). Interestingly, PKA directly phosphorylates and inhibits AMPK (Hurley et al., 2006; Berdeaux et al., 2007; Mihaylova et al., 2011), which can be prevented by 1 µM H-89 (Djouder et al., 2010). Furthermore, chronic high [Ca^2+^] depresses AMPK activity in a CaMKII-dependent manner. Inhibition of CaMKII by KN-93 eliminates the negative effect of high [Ca^2+^] on AMPK (Park et al., 2011). Increase in cytosolic [Ca^2+^] also can activate CAMK kinase 2 (CAMKK2, also known as CAMKKβ), which subsequently activates AMPK by phosphorylation at Thr172 (Hawley et al., 2005; Hurley et al., 2005; Woods et al., 2005; Fogarty et al., 2010; Mihaylova and Shaw, 2011). Consideration of these pathways initiated downstream of µsPEF exposure leads us to postulate the protein kinase-based mechanisms by which HDAC4 and HDAC5 shuttle between the cytoplasm and nucleus (Figure 9).

Other kinases are likely at play in the regulation of µsPEF exposure-induced nucleocytoplasmic shuttling of HDAC4 and HDAC5. Both active CaMKII and PKD are required for HDAC5 nuclear export within rat hippocampal neurons stimulated by ketamine (Choi et al., 2015). CaMK regulates 14–3-3 binding to HDAC5 *via* phosphorylation of either Ser-259 or Ser-498, enabling dissociation from MEF2 and export of HDAC5 from the nucleus (McKinsey et al., 2000a; McKinsey et al., 2001). Further work would need to be done to test how PEF exposure alters the structure and binding interactions among protein kinases and class IIa HDAC’s.

According to our results, we propose the model shown in Figure 9C of kinase regulation of HDAC4 and HDAC5 localization within MCF7 upon µsPEF exposure. Exposed MCF7 cells have a higher HDAC4 N/C ratio but not a significantly changed HDAC5 N/C ratio when compared to unexposed controls in either solution (Figures 5, 6). HDAC4 localization occurs without hetero-oligomerization with HDAC5 (Backs et al., 2006; Backs et al., 2008) in MCF7 exposed to this dosage of µsPEF. Since H-89 treatment plus µsPEF exposure maximizes the N/C ratio measured, the nuclear export of HDAC4 mediated by AMPK and perhaps PKA (or another basophilic kinase) is inactivated by µsPEF exposure. Treatment with KN-93 inhibits CaMKII-mediated nuclear exclusion of HDAC4 but more importantly could block some voltage-gated ion channels, lowering the peak [Ca^2+^]_i_ elicited by µsPEF exposure, while also enabling more active AMPK export of HDAC4 from the nucleus. The HDAC4 N/C ratio in MCF7 cells exposed to µsPEF in the presence of KN-93 is significantly lower than that for µsPEF exposure alone. Thus, AMPK-mediated (and perhaps PKA-mediated) nuclear export of HDAC4, which is mitigated following µsPEF exposure, appears to dominate intracellular HDAC4 localization within MCF7 breast cancer cells. Finally, HDAC5 localization is affected by KN-93 and H-89, more so in SOS, suggesting CaMKII contributes to nuclear exclusion of HDAC5 (without hetero-oligomerization with HDAC4) in MCF7 cells exposed to µsPEF.

CHO-K1 cells respond differently to µsPEF exposure that MCF7 breast cancer cells, as captured by our model in Figure 9D of kinase regulation of HDAC4 and HDAC5 localization within CHO-K1 cells. The kinase regulation of HDAC4 and HDAC5 localization within CHO-K1 cells seems opposite to that of MCF7 cells. Hetero-oligomerization of HDAC4 with HDAC5 imparts regulation by CaMKII to HDAC5 localization (Backs et al., 2006; Backs et al., 2008), which we observe in CHO-K1 cells. Exposure of CHO-K1 cells in SOS leads to lower N/C ratios of both HDAC4 and HDAC5 when compared to unexposed controls (Figures 5, 6). Compared to µsPEF exposure alone, combination of µsPEF exposure with KN-93 treatment causes significant increases in the N/C ratios of HDAC4 in both solutions and of HDAC5 in SOS. Treatment of CHO-K1 with H-89 plus µsPEF exposure significantly changes the N/C ratio from that of µsPEF exposure only for HDAC5 in SOS. These trends indicate that CaMKII dominates exclusion of HDAC4 and HDAC5 from the nuclei of CHO-K1 cells, with a smaller contribution from a kinase inhibited by H-89 to HDAC5 localization. As noted above, other [Ca^2+^]_i_-responsive enzymes such as CAMKK2, which activates AMPK (Hawley et al., 2005; Hurley et al., 2005; Woods et al., 2005; Fogarty et al., 2010; Mihaylova and Shaw, 2011), could affect the observed µsPEF exposure-induced HDAC4 and HDAC5 translocation patterns in CHO-K1 cells. The lower levels of peak [Ca^2+^]_i_ within CHO-K1 cells, with their dearth of voltage-gated ion channels, as compared to peak levels in MCF7 cells highlights how electropermeabilization from a given dose of µsPEF exposure can activate an enzyme, e.g., CaMKII, in one type of cell but inactivate another enzyme, e.g., AMPK, in another type of cell for opposing outcomes.

In summary, the differential responses of the human breast cancer cell line, MCF7, and the hamster ovary cell line, CHO-K1, imply targeted, cell-specific effects to µsPEF exposure can be exploited. With regards to HDAC4 and HDAC5 localization in response to µsPEF exposure, AMPK and PKA activity plays an important role in MCF7 cells, whereas CaMKII activity appears crucial in CHO-K1 cells. The proposed models illustrating kinase-based responses of cells to µsPEF exposure (Figures 9, 10) do not represent generic responses of mammalian cells to high cytosolic [Ca^2+^]. Different types of cells respond uniquely to these levels of µsPEF exposure. Further work is needed to fully elucidate the mechanisms linking the physical µsPEF-based stimulation and the observed intracellular responses vis-à-vis HDAC4 and HDAC5. Class IIa HDAC localization bears importance in cell fate outcomes, such as cell proliferation (Clocchiatti et al., 2011; Guan et al., 2012) and cell death (Backs et al., 2011). Although HDAC expression levels within CHO-K1 cells do not appear readily available in the literature (Christensen et al., 2018), HDAC4 and HDAC5 are transcriptionally upregulated in urothelial cancer cells versus normal urothelial cells (Niegisch et al., 2013). Levels of HDAC4 and HDAC5 have been reported as transcriptionally overexpressed (Ozdag et al., 2006; Patani et al., 2011) in human breast cancer compared to normal or benign breast tissue. Future experiments are warranted to determine if the differential responses of HDAC nucleocytoplasmic shuttling induced by µsPEF exposure in cells translates to selective treatment of tumor cells.

## Data Availability

The raw data supporting the conclusion of this article will be made available by the authors, without undue reservation.
