# Supplementary material for: Histone deacetylase 4 and 5 translocation elicited by microsecond pulsed electric field exposure is mediated by kinase activity
Source: Front Bioeng Biotechnol. 2022 Nov 17;10:1047851. doi: 10.3389/fbioe.2022.1047851 (PMC9713944; doi:10.3389/fbioe.2022.1047851)
Supplement: Supplementary file 1 [file Table1.DOCX]

***Supplementary Material***

**Supplementary Table S1.** Microscopy image acquisition and processing steps.

| **Image Acquisition** | **Image Positioning** | |
| --- | --- | --- |
| The sample is placed onto the inverted confocal microscope stage, and the focal plane is finely adjusted onto the cells. Multiple images are captured in different locations within each petri dish to ensure the best images representing a sufficient number of cells within the sample are available for processing. Looking at pictures to the right, at least one image at each of five locations within a dish are acquired. The order of capturing images within the green rectangles starts in the middle, then the upper, lower, left, and right. | 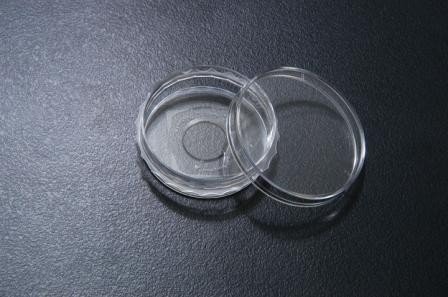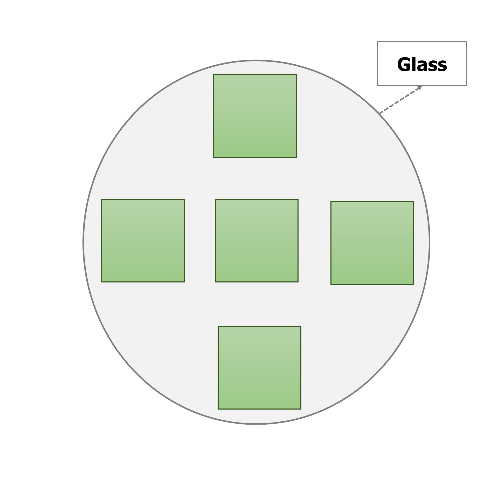 | |
| **Image Processing Steps** | **HDAC4 (Immunofluorescence)** | **NUCLEUS (Propidium Iodide)** |
| 1. Open a raw image and automatically enhance brightness and contrast. Save the resulting image. | 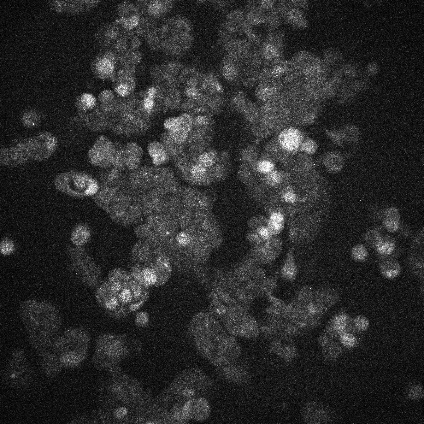 | 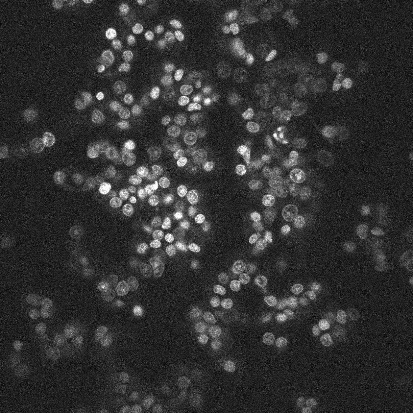 |
| 1. Produce a Gaussian blur image with sigma = 500 from the opened raw image. Save the Gaussian blur image. | 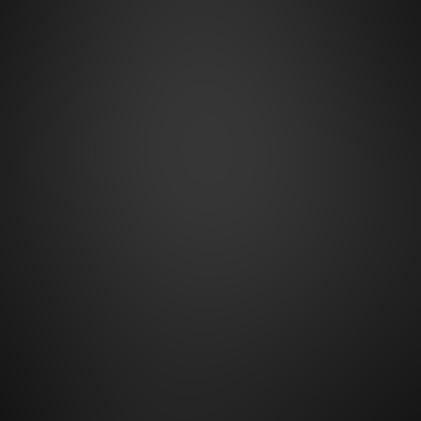 | 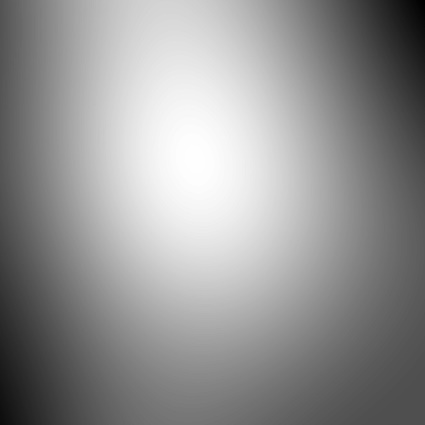 |
| 1. Subtract the Gaussian blur image from the enhanced contrast image. Save the resulting background-corrected image. 2. Measure total intensity (raw integrated density) of the background-corrected image of HDAC immunofluorescence. | 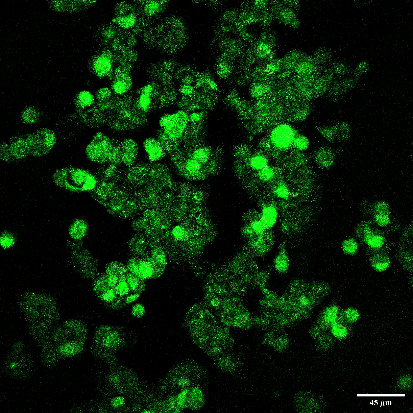 | 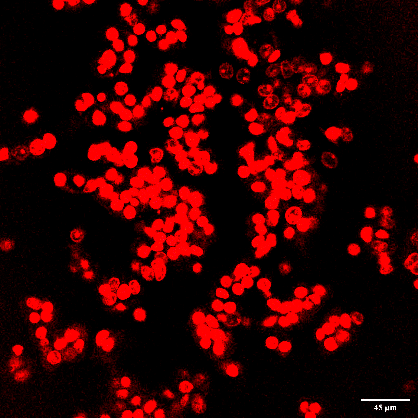 |
| 1. Create a binary mask image from the image of stained nuclei. Save the binary mask image and select it. 2. Redirect measurement of intensity from the selected binary mask image onto the corresponding background-corrected HDAC immunofluorescence image. In other words, measure raw integrated density of HDAC within the nuclei regions of interest (ROI). | 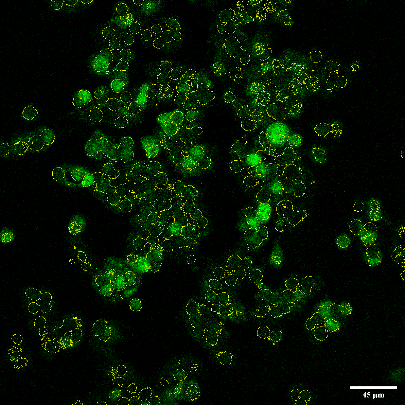 |  |
| 1. Calculate the Nuclear-to-cytoplasmic (N/C) ratio:   HDAC in nucleus = Raw integrated density of HDAC in nuclei ROI  HDAC in cytoplasm = (Raw integrated density of total HDAC) - ( Raw integrated density of HDAC in nuclei ROI)  N/C ratio = (HDAC in nucleus) / (HDAC in cytoplasm) | | |

**Supplementary Table S2.** Number of images analyzed for each experimental condition.

| **Number of images per condition** | | | | | |
| --- | --- | --- | --- | --- | --- |
| Cell Line + Condition | | HDAC4 | | HDAC5 | |
|  |  | CAF | SOS | CAF | SOS |
| MCF7 + Pulse | Control | 6 | 6 | 7 | 7 |
|  | 1 P | 7 | 7 | 5 | 7 |
|  | 10 P | 8 | 6 | 6 | 4 |
|  | 30 P | 8 | 6 | 8 | 7 |
|  | 50 P | 8 | 9 | 7 | 7 |
| CHO-K1 + Pulse | Control | 6 | 6 | 6 | 5 |
|  | 1 P | 5 | 6 | 6 | 4 |
|  | 10 P | 6 | 6 | 6 | 6 |
|  | 50 P | 5 | 6 | 8 | 8 |
| MCF7 + Inhibitor | KN-93 | 6 | 6 | 5 | 5 |
|  | H-89 | 6 | 6 | 6 | 6 |
| CHO-K1 + Inhibitor | KN-93 | 5 | 5 | 5 | 5 |
|  | H-89 | 6 | 6 | 6 | 6 |


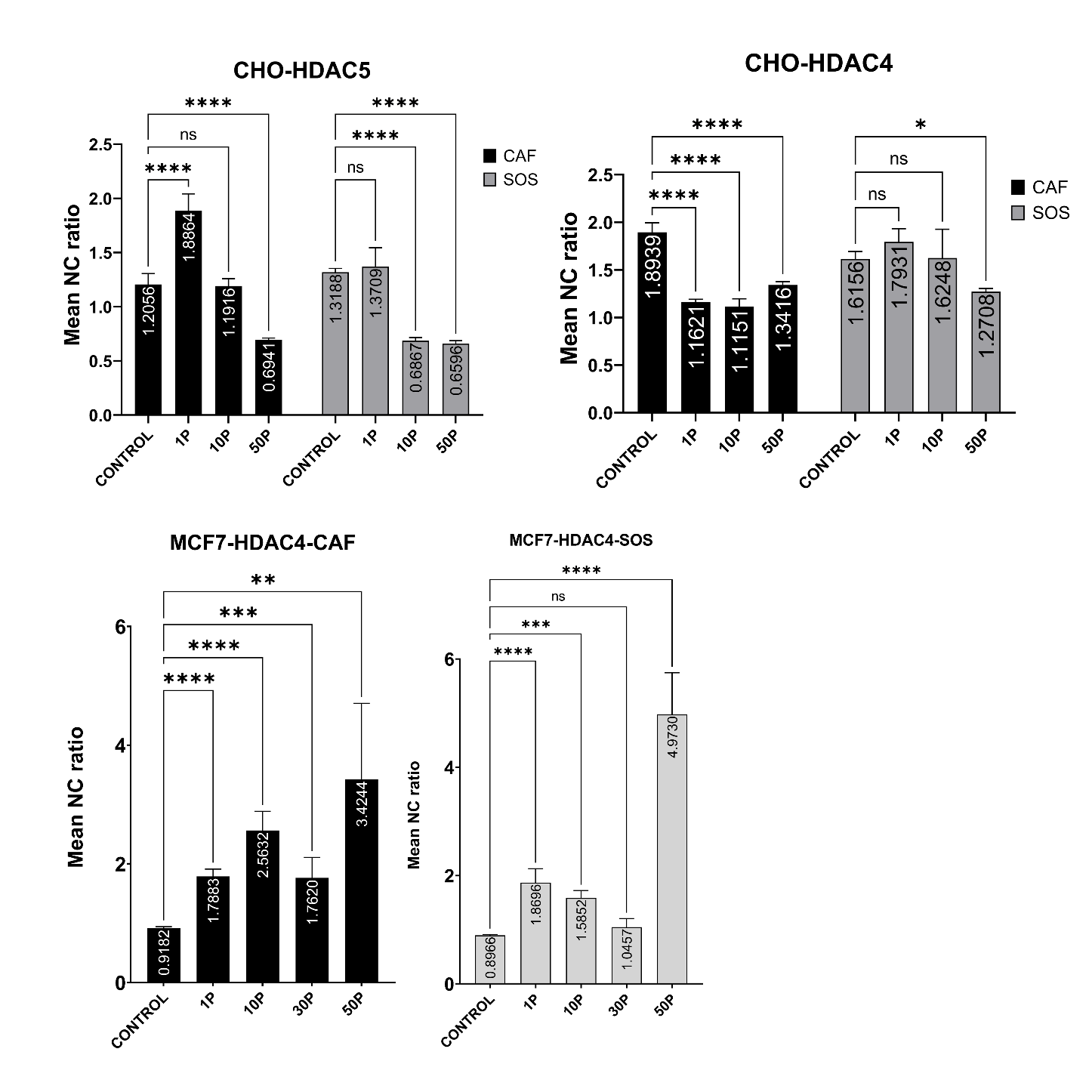

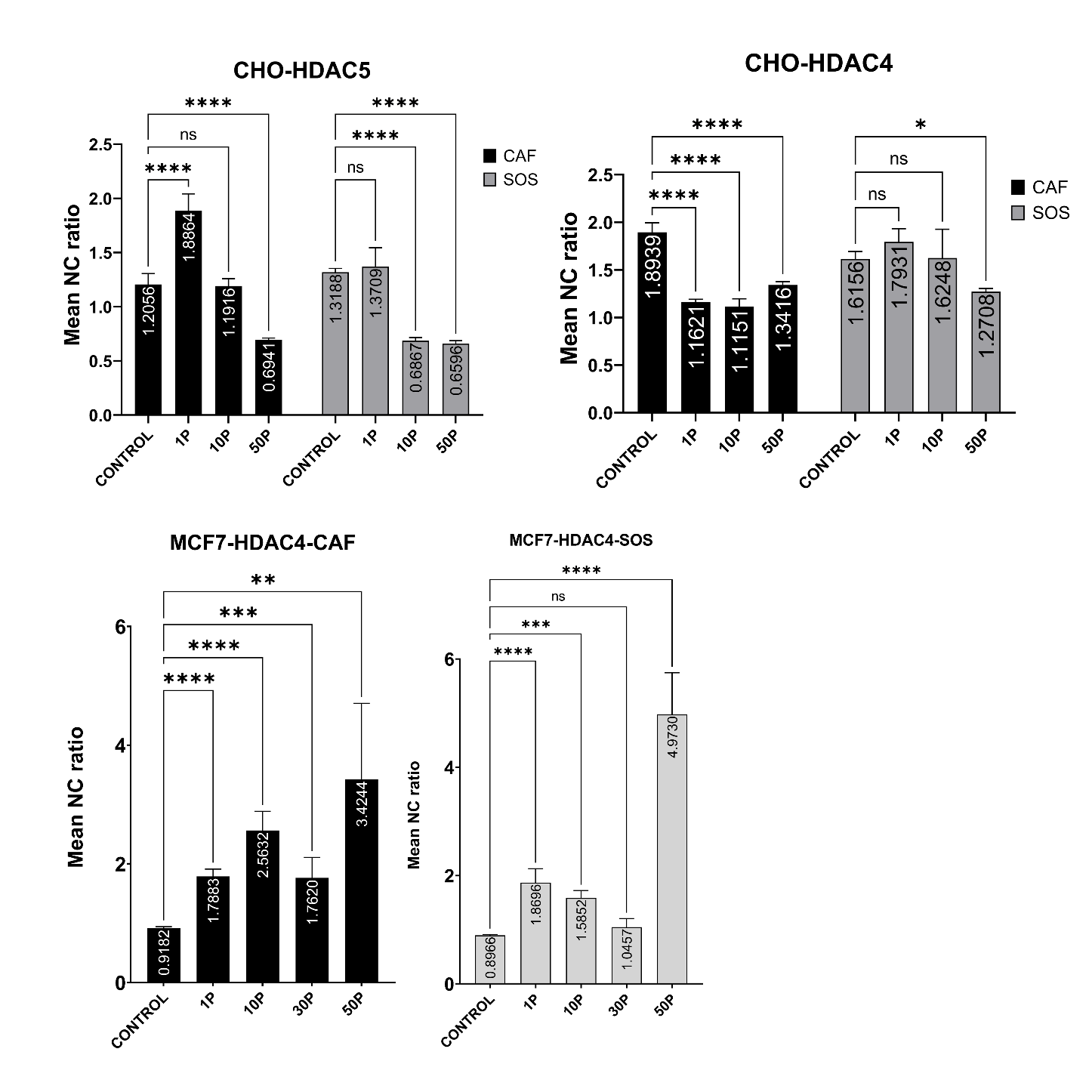


**Supplementary Figure S1.** Results from preliminary experiments showing HDAC4 translocation within MCF7 (top) and CHO-K1 (bottom) and cells elicited by µsPEF exposure in either CAF (dark) or SOS (light). These data represent different IFA processing methods than those presented within the main manuscript. Specifically, the blocking solution contained a lower concentration of BSA (~ 0.1 wt%). NC ratio values shown here differ from those shown in Figure 2. However, trends in differences among mean N/C ratios of HDAC4 in sham controls and µsPEF-exposed samples shown here are similar to the results from final experiments shown in Figure 2. Each sample was exposed to 0 (control), 1, 10, 30 or 50 consecutive pulses, P, of 100 μs duration, 1.45 kV/cm and a repetition rate of 1 Hz. Data represent 4 – 9 images from one dish per condition. Error bars represent one standard deviation. Statistical significance tested by ANOVA is indicated as (ns) p < 0.1234, *p < 0.0332, **p < 0.0021, ***p <0.0002 and ****p <0.0001.
